# Identifying diseases that cause psychological trauma and social avoidance by GCN-Xgboost

**DOI:** 10.1186/s12859-020-03847-1

**Published:** 2020-12-16

**Authors:** Huijuan Xu, Hairong Wang, Chenshan Yuan, Qinghua Zhai, Xufeng Tian, Lei Wu, Yuanyuan Mi

**Affiliations:** 1 First Department of Breast Surgery, Shanxi Provincial Cancer Hospital, Taiyuan, People’s Republic of China; 2 Department of Nursing, Shanxi Provincial Cancer Hospital, Taiyuan, People’s Republic of China; 3 Department of Nutrition, Shanxi Provincial Cancer Hospital, Taiyuan, People’s Republic of China; 4 Department of Medical Records, Shanxi Provincial Cancer Hospital, Taiyuan, People’s Republic of China; 5 Second Department of Breast Surgery, Shanxi Provincial Cancer Hospital, Taiyuan, People’s Republic of China

**Keywords:** Graph convolutional network, Xgboost, Psychological trauma, Breast cancer

## Abstract

**Background:**

With the rapid development of medical treatment, many patients not only consider the survival time, but also care about the quality of life. Changes in physical, psychological and social functions after and during treatment have caused a lot of troubles to patients and their families. Based on the bio-psycho-social medical model theory, mental health plays an important role in treatment. Therefore, it is necessary for medical staff to know the diseases which have high potential to cause psychological trauma and social avoidance (PTSA).

**Results:**

Firstly, we obtained diseases which can cause PTSA from literatures. Then, we calculated the similarities of related-diseases to build a disease network. The similarities between diseases were based on their known related genes. Then, we obtained these diseases-related proteins from UniProt. These proteins were extracted as the features of diseases. Therefore, in the disease network, each node denotes a disease and contains the information of its related proteins, and the edges of the network are the similarities of diseases. Then, graph convolutional network (GCN) was used to encode the disease network. In this way, each disease’s own feature and its relationship with other diseases were extracted. Finally, Xgboost was used to identify PTSA diseases.

**Conclusion:**

We developed a novel method ‘GCN-Xgboost’ and compared it with some traditional methods. Using leave-one-out cross-validation, the AUC and AUPR were higher than some existing methods. In addition, case studies have been done to verify our results. We also discussed the trajectory of social avoidance and distress during acute survival of breast cancer patients.

## Background

When people experience some sudden diseases, catastrophic injury or sexual violence, they are very likely to get post-traumatic stress disorder (PTSD) [1] which is a series of mental disorders. PTSD is a kind of delayed psychogenic response which is hard to overcome. It has been reported that the prevalence of PTSD [2] is about 2.5%. PTSD can be caused by many sudden, catastrophic, or threatening accidents such as traffic accidents, wars, diseases, death of close friends.

Due to the high development of medical care, various diseases can be treated now. Patients not only need increase survival time and rate, but also a health mind. It has been reported that patients who are survival from major diseases are at high risk of mental problems. These mental problems are neurosurgical diseases [3, 4] which give their families and society heavy burdens. Breast cancer which is the most frequent malignancy in women is a kind of these traumatic diseases [5]. The comment treatments such as mastectomy and chemotherapy are very likely to cause psychosocial, mental, and economic problems. If these problems are not addressed effectively, not only the self-esteem and quality of life would be affected, but also the survival time [6]. Researchers have found people are afraid of joining social activities, and they are prone to depression after experiencing breast cancer. This is mainly caused by the loss of femininity, which may lead to low self-esteem and pessimism.

Studies also point out the benefits of trauma. Tedeschi and Calhoun [7] developed a novel concept named ‘Posttraumatic Growth (PTG)’. They found some negative emotions sometimes can give people positive psychological changes.

However, researchers mainly focused on diseases and ignored the PTSA brought by diseases [8]. At least, we should know the diseases that can cause PTSA. However, finding this kind of diseases needs investigate hundreds of patients, which is time and money consuming. Therefore, in this paper, we developed a computational method to identify diseases that cause PTSA based on disease similarity. More and more studies have found that similar diseases are usually caused by similar molecules [9, 10]. Therefore, they can be diagnosed by similar biomarkers or phenotypes, and can be cured by similar drugs. In this paper, we put forward a hypothesis: similar diseases may cause similar psychological problems. In 2004, Freudenberg and Propping obtained phenotypes of diseases from the Online Mendelian Inheritance in Man (OMIM) and used them to calculate the similarity of diseases. In recent years, the number of phenotypes is increasing, which prompted researchers to develop more methods to measure disease similarity at phenotypic level. Due to the rapid development of sequencing technology, measuring disease similarity based on molecule is popular now. Many researchers have calculated diseases similarity based on genes. mRNA expression data and protein interactions were used to calculate disease similarities by Suthram et al. [11]. Cheng et al. [12] developed ‘SemFunSim’ method which considered gene functional network to calculate disease similarities.

Deep learning methods are widely used in the field of bioinformatics [13–17] nowadays. Since we could build a disease network, we used Graph Convolutional Network (GCN) [18] to extracted features from network. Finally, we could identify diseases that cause PTSA by Xgboost.

## Results

### Data description

**Fig. 1:**
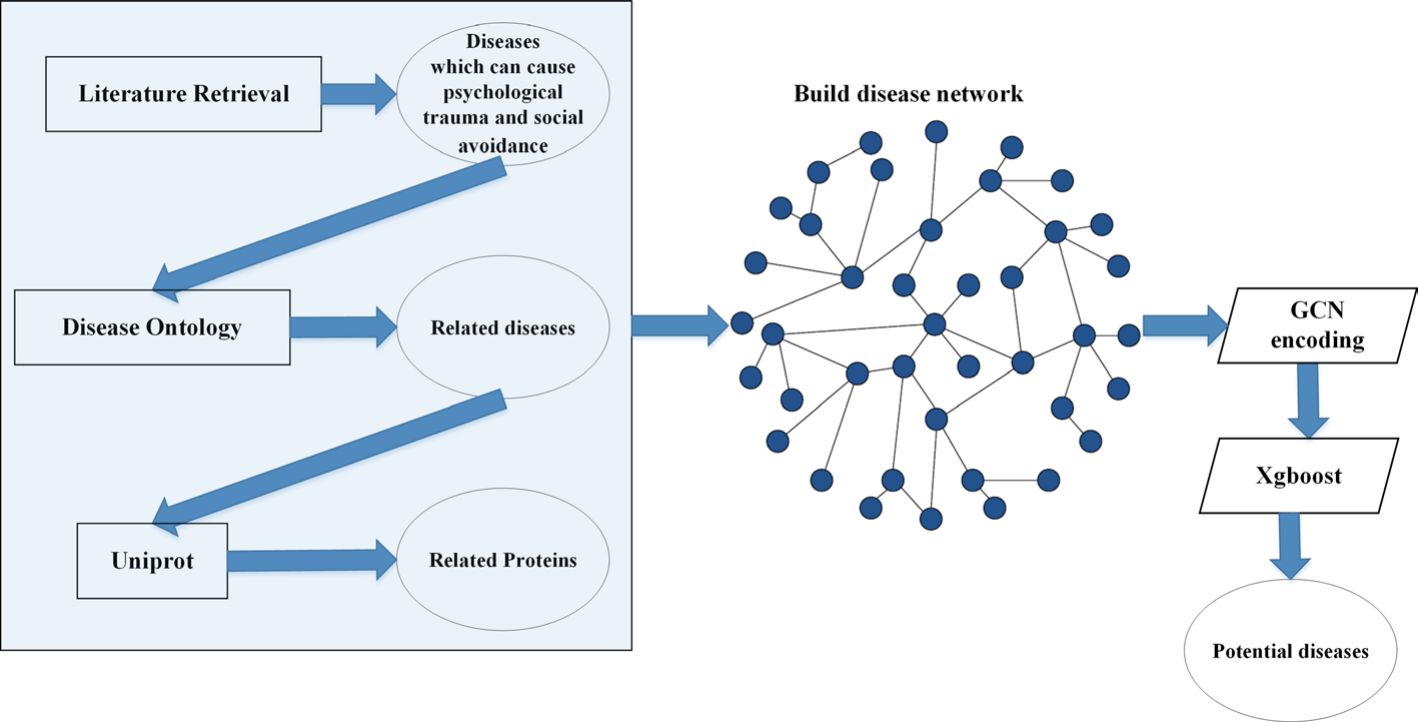
Comparison results

Firstly, we draw Fig. 1 to show the similarity of diseases. As we can see in Fig. 1, 66% of all similarities are lower than 0.1. Only a few of similarities are higher than 0.5.

**Fig. 2:**
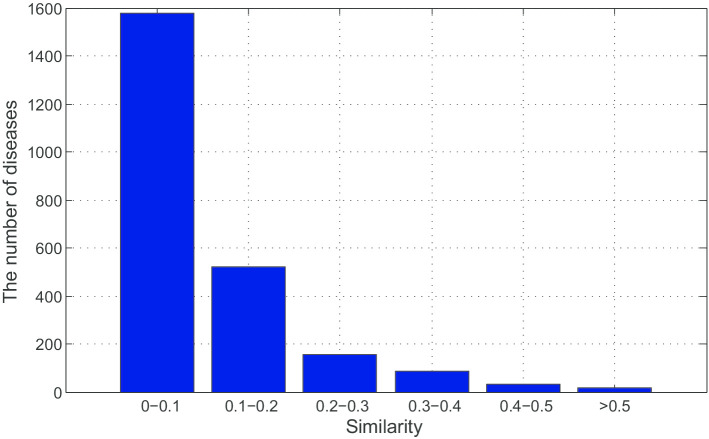
Framework of GCN-Xgboost

As shown in Fig. 2, some proteins are related to more than 1000 diseases, whereas some proteins are only associated with less than 100 diseases. Therefore, the features are sparse.

### Comparison experiments

Since only 23 diseases are known to cause PTSA, we used leave-one-out cross validation to test the performance of GCN-Xgboost. We divided all diseases into 23 groups. For each time, we used one known disease with one group of unknown diseases as the test dataset and the rest are the training set.

We compared our method with support vector machine (SVM), artificial neural network (ANN), deep neural network (DNN) and random forest (RF). Figure 4 shows the AUC and AUPR of the results.

**Fig. 3:**
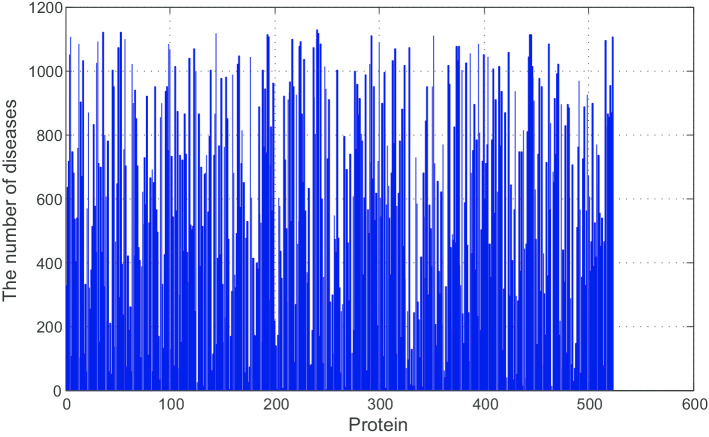
Distribution of disease similarity

As we can see from Fig. 3, GCN-Xgboost performed best among these five methods with AUC 0.97 and AUPR 0.78. The second best method is DNN, since it can learn complex non-linear relationship from sparse data. SVM is the worst since it can not handle high dimensional features.

### The power of GCN

Although GCN-Xgboost performed best among these methods, we still want to know the reason. Therefore, we only used Xgboost to identify diseases which can cause PTSA and compared the results with GCN-Xgboost’s.

The results are shown in Table 1.

**Table 1:** Comparison between GCN-Xgboost and Xgboost

	Dataset 1	Dataset 2
GCN-Xgboost	0.97	0.78
Xgboost	0.96	0.61

As we can see in Table 1, the AUC did not change much after using GCN, but the AUPR changed a lot. The AUPR of Xgboost was only 0.61, but GCN-Xgboost was 0.78, which means GCN-Xgboost can reduce false positive. Since GCN encoded the similarities of diseases, more information were provided so the method can perform better.

### Case study

After verifying the effectiveness of GCN-Xgboost, we used it to identify diseases which can cause PTSA. Therefore, all the positive diseases are used as the positive samples. We randomly selected 100 unknown diseases as negative samples to built the model. We found 228 diseases were identified as diseases that cause PTSA.

To verify whether our results are correct, we searched literatures to do case study.

Flatt et al. [19] reported that Alzheimer’s disease is very likely to cause PTSD. In addition, they also found people with PTSD and depression have twice the risk of dementia.

Yi-Frazier et al. [20] found that families and individuals of adolescents with type 2 diabetes are experiencing significant psychological stress.

### PTSA in breast cancer

Breast cancer patients are at high risk of PTSA, which is a well-known fact.

From February 2017 to October 2017, 200 eligible patients with breast cancer were selected by randomly sampling from Department of Breast Surgery at the Shanxi Provincial Tumor Hospital. After obtaining written informed consent, trained researchers fill out the questionnaire for each patient.

All selected patients meet the following four conditions: (1) patients with breast cancer are diagnosed by pathological examination and are agreed to mastectomy; (2) age ≥ 18 years; (3) all the patients have received primary school or higher education and are able to communicate effectively; (4) they are awareness of diagnosis and voluntary participation.

Patients are excluded if they meet one of the following 4 conditions: (1) they have complications, such as heart disease, hypertension, and kidney disease; (2) they have other malignancies; (3) they are receiving antipsychotics for mental disorders.

The questionnaire includes: (1) basic information: age, occupation, education, retirement status, payment method for medical care, marital status, religion, and menopause status; (2) disease-related data: breast volume, severity of alopecia, breast cancer family history, and willingness of contralateral prophylactic mastectomy; (3) basic information of spouse: age, nationality, religion, education, occupation, and retirement status.

The Social Avoidance and Distress Scale (SADS) [21] was developed by Watson and Friend in 1969 which consists of 14 items measuring social avoidance and 14 items measuring social distress. Each item can be answered by “yes” or “no”. The reliability of the avoidance and distress scales are 0.87 and 0.85, respectively. Scores for each item are summed to obtain a total score. If the total score is higher than 9, the patients are suffering social avoidance and distress. The total score for healthy individuals in China is 8.03 ± 4.86.

The Self-Esteem Scale (SES) developed by Rosenberg in 1965 is composed by 10 items. The items are rated by a four-point scale, where 1 = strongly agree, 2 = agree, 3 = disagree, and 4 = strongly disagree. Therefore, the total score ranges from 10 to 40. If the total score is lower than 25, the patient is low self-esteem. 26–32 represents moderate self-esteem, and 33 or higher represents high self-esteem. It is the most commonly used instrument to measure self-esteem in China.

Alopecia was graded according to National Cancer Institute Common Terminology Criteria for Adverse Events (NCI-CTCAE) 4.0 (grade 0: no alopecia; grade 1: hair loss < 50%, which is only visible close by and may need to be covered by different hairstyle; grade 2: hair loss > 50%, which needs to be covered by wigs or hats.

Breast volume was defined as brassiere cup size, i.e., the difference between the upper and lower chest circumferences. The cup size was recorded as A to E.

Considering that the number of patients will decrease during follow-up, the sample size was increased by 20%. A total of 800 questionnaires were distributed in four rounds of surveys.

Four rounds of face-to-face survey were conducted by trained researchers. Patients are divided into 4 groups based on the four phases of treatment: (1) after diagnosis but before mastectomy, (2) after mastectomy but before chemotherapy, (3) at mid-chemotherapy (in the second cycle), (4) at the end of chemotherapy. A total of 192 patients completed all the four rounds of survey and a total of 768 valid questionnaires were collected.

As shown in Table 2, results from the questionnaires showed significant differences in scores among the four phases of acute survival. The mean score of the four phases was 12.87 ± 5.71, which was significantly higher than that for healthy individuals in China (t = 11.741, *P* < 0.001).

**Table 2:** Comparison of social avoidance and distress scores in the four phases

	Social avoidance	Social distress	Total score
Before mastectomy	6.26 ± 3.59	6.39 ± 3.98	12.65 ± 7.31
After mastectomy but before chemotherapy	7.46 ± 3.78	6.92 ± 3.54	14.39 ± 6.85
Mid-chemotherapy	6.38 ± 3.39	6.52 ± 3.58	13.18 ± 6.76
End of chemotherapy	5.31 ± 2.90	6.22 ± 3.89	11.80 ± 6.07
Z	13.746	27.156	20.647
*P*	0.003	< 0.001	< 0.001

As shown in Table 3, statistical analysis revealed significant differences in self-esteem among the four phases of acute survival (Table 3). Among patients with low self-esteem, the number of patients after mastectomy but before chemotherapy was the largest (28.1%). Since then, the number of patients with low self-esteem has decreased, while the number of patients with moderate self-esteem has increased.

**Table 3:** Self-esteem changes in the four phases

	Stage 1	Stage 2	Stage 3	Stage 4
*Low self-esteem*				
Cases	9	54	28	8
Percentage	4.7	28.1	14.6	4.2
*Moderate self-esteem*				
Cases	172	130	152	177
Percentage	89.6	67.6	79.2	92.2
*High self-esteem*				
Cases	11	8	12	7
Percentage	5.7	4.2	6.2	3.6
X^*2*^	66.870			
*P*	< 0.001			

The results of univariate analysis of social avoidance and distress are shown in Table 4. Breast size, willingness for contralateral prophylactic mastectomy, self-esteem, and spouse education are factors that cause significant differences in social avoidance and suffering.

**Table 4:** Univariate analysis of social avoidance and distress

Factor	Social avoidance (%)	No social avoidance (%)	X^*2*^	*P* value
*Breast size*				
A cup	58.8	37.9	12.4	0.006
B cup	14.7	33.1		
C cup	13.2	21.0		
D + E cup	13.2	8.1		
*Willingness for contralateral prophylactic mastectomy as gene mutation carriers*				
Yes	73.5	46.8	12.7	< 0.001
No	26.5	53.2		
*Self-esteem*				
Low	35.3	2.4	34.2	< 0.001
Moderate	64.0	81.0		
High	0.7	16.7		
*Spouse education*				
Primary school	26.7	11.9	7.92	0.048
Junior high school	38.0	33.3		
Senior high school/technical secondary school	31.0	31.0		

For multivariate analysis, variables are defined as follows: breast size: 0 = A cup, 1 = B cup, 2 = C cup, 3 = D + E cup; spouse education: 1 = primary school and below, 2 = junior high school, 3 = senior high school/technical secondary school, 4 = university and above; self-esteem scale: 0 = low, 1 = moderate, 2 = high; and willingness for contralateral prophylactic mastectomy as gene mutation carriers: 1 = yes, 0 = no. The results are shown in Table 5.

**Table 5:** Multivariate logistic regression analysis of social avoidance and distress

Variable	B	S.E	Wald	*P*	OR
A cup			7.464	0.058	
B cup	− 0.852	0.472	3.254	0.071	0.427
C cup	− 0.323	0.514	0.394	0.530	0.724
D + E cup	0.882	0.599	2.164	0.141	2.415
Spouse education—primary school and below			5.231	0.156	
Spouse education—junior high school	− 0.356	0.455	0.613	0.434	0.700
Spouse education—senior high school/technical secondary school	− 1.033	0.524	3.890	0.049	0.356
Spouse education—university and above	− 1.042	0.630	2.741	0.098	0.353
Self-esteem low			19.271	0.001	
Self-esteem—moderate	− 1.740	0.396	19.271	0.001	0.176
Self-esteem high	− 21.639	13,730	0.000	0.999	0
Willingness for prophylactic mastectomy	0.831	0.385	4.662	0.031	2.297
Constant term	0.823	0.558	2.173	0.140	2.277

Compared with spouses with elementary education and below, spouses with high school/technical education are the protective factors to avoid social avoidance. Compared with low self-esteem, moderate self-esteem is a protective factor to avoid social avoidance. The willingness of contralateral preventive mastectomy in genetic mutation carriers is a risk factor for social avoidance.

## Discussion

Breast cancer patients experience severe social avoidance and distress during acute survival, especially in the stage between mastectomy and chemotherapy. Mastectomy can induce psychological and physical stress. Moreover, the loss of femininity after the operation exacerbated the distress. Breast loss and hair loss, nausea and weakness caused by chemotherapy seriously affect the mood of patients. They may even worry about being disliked by others, thus avoiding social interaction. Medical staff should cooperate with patients’ families to understand and support patients, create a relaxed and positive environment for them, and enhance their sense of family and social belonging.

Self-esteem is a person’s self-emotional experience and evaluation in the social process. It is the core of self-awareness and an important indicator of mental health. Self-esteem affects patients' cognition, emotion, behavior, and mental health. In this study, in the period between mastectomy and chemotherapy, the number of patients with the highest inferiority complex was the largest. This may be related to the decline in self-care ability, self-identity disorder and weakened social role function. Patients tend to avoid social interactions, become more sensitive to interpersonal relationships, anxious and distressed. Self-esteem is a protective factor for mental health. An optimistic and positive attitude towards reality can enhance resilience. Medical staff should share successful cases of successful fight against diseases and recommend breast reconstruction and rehabilitation to help patients with low self-esteem improve their self-emotional experience and evaluation, and encourage them to express their emotions.

It has been suggested that the spouse’s concern about the patient’s appearance is an important factor in postoperative depression. The negative emotions of the spouse will further increase the psychological burden of the patient. The support of the spouse can provide positive psychological support for the patient. The results of this study indicate that the education level of the spouse may be related to social avoidance. A well-educated spouse may help patients understand and deal with the disease correctly, choose the best treatment plan, and provide them with positive psychological support to reduce their negative emotions. Therefore, medical staff should provide the spouses of breast cancer patients with necessary psychological and information support, improve their ability to care for the patients, and encourage and support the patients to reduce the patients' social avoidance.

The results of this study indicate that Contralateral preventive mastectomy for genetic mutation carriers increases the possibility of avoiding social interaction or aggravates social distress. According to reports, patients with unilateral breast cancer have an increased risk of contralateral breast cancer by 0.5–0.75% each year. Contralateral mastectomy has been shown to be effective for genetic mutation carriers. In this study, 56.25% of subjects were willing to undergo contralateral prophylactic mastectomy. However, this is a risk factor that society avoids and troubles. Loss of bilateral breasts, surgical trauma, increased risk of complications, and financial burden lead to fear, anxiety and depression.

To sum up, medical staff should pay attention to the psychological changes of breast cancer patients during the entire acute survival period, especially after mastectomy and the middle period of chemotherapy, and provide them with positive psychological support. Medical staff are obliged to help patients improve self-evaluation, promote psychological adjustment and enhance anti-stress ability. In addition, although contralateral preventive mastectomy can effectively prevent breast cancer, it may increase psychological and physical trauma, cause or increase social avoidance and distress, and reduce the patient's quality of life. Therefore, contralateral prophylactic mastectomy should only be performed under strict indications to avoid excessive aggressive treatment.

## Conclusions

PTSA seriously threats patients’ mental health and gives burden on the society. With the advancement of medical technology, patients are not only satisfied with the physiological cure, but also the psychological cure. PTSA is related to the quality of life of the patients after treatment. Therefore, special care is needed for patients with diseases that may cause PTSA. To achieve personalized treatment, we should know the diseases can cause PTSA at first. However, investigating hundreds of patients for each disease is time and money consuming. Therefore, in this paper, we developed ‘GCN-Xgboost’ to identify diseases that cause PTSA.

First, we calculated the similarities of diseases based on their related genes. Then, we obtained their related proteins from UniProt. Then, a disease network was built. GCN was used to encode the network to extract features for each disease. After encoding, the feature of each disease not only contains their related proteins, but also their relationship with other diseases. Finally, Xgboost was used to build model to identify diseases that cause PTSA.

We verified our method by cross-validation and compared our method with other existing methods. After verifying the effectiveness of our method, we did case studies to verify the accuracy of our results. At last, we discussed the PTSA in breast cancer.

## Methods

### Work flow

**Fig. 4:**
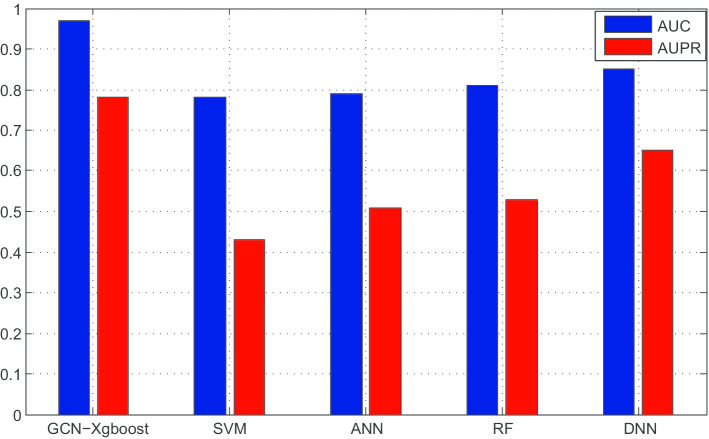
Distribution of disease-related protein

Figure 4 shows the work flow of our method. Firstly, we searched diseases that cause PTSA in PubMed. Then, Disease Ontology (DO) [22] was used to obtain these diseases-related diseases. After that, gene-based similarity calculation method was used to calculate the similarities of all the obtained diseases. Then, we could build a disease network based on the disease similarities. Secondly, we obtained each disease-related proteins from Uniprot [23] and we encoded these proteins to be the features of diseases. Then, each node in the disease network also contains information about its protein. Then, GCN was used to extract features from disease network. Finally, Xgboost was used to do the classification. We labeled known diseases that cause PTSA as 1, unknown diseases as 0.

### Calculating disease similarity

Most of the diseases are associated with genes. Therefore, we calculated the similarity of diseases based on genes. We obtained disease-related genes by HumanNet [24]. Each gene interaction has a log likelihood score (LLS). Firstly, we need to normalize them.

1 $$LLS_{N} (g_{i} ,g_{j} ) = \frac{{LLS(g_{i} ,g_{j} ) - LLS_{Min} }}{{LLS_{Max} - LLS(g_{i} ,g_{j} )}}$$

$g_{i} ,g_{j}$ denotes i_th_ and j_th_ gene respectively. $LLS_{N} (g_{i} ,g_{j} )$ is the LLS after normalization.

Therefore, the functional similarity score of two bunches of genes could be calculated by:

2 $$sim(g_{i} ,g_{j} ) = \left\{ {\begin{array}{*{20}l} 1 \hfill & {i = j} \hfill \\ {LLS_{N} (g_{i} ,g_{j} )} \hfill & {e(i,j) \in (HumanNet)} \hfill \\ 0 \hfill & {e(i,j) \notin (HumanNet)} \hfill \\ \end{array} } \right.$$

$e(i,j) \in (HumanNet)$ means the interaction edge between $g_{i} \;and\;g_{j}$ is included in the HumanNet.

Then, if we want to calculate the association between one gene g and a gene set $G = \{ g_{1} ,g_{2} , \ldots ,g_{k} \}$, we could use Eq. 3.

3 $$F(G,g) = \mathop {\max }\limits_{1 \le i \le k} (sim(g,g_{j} ))\;\;,g_{j} \in G$$

k denotes the number of genes in G.

Finally, two diseases could be considered as two gene sets $G_{1}$ and $G_{2}$. Therefore, the similarity between two diseases could be calculated as following:

4 $$sim(G_{1} ,G_{2} ) = \frac{{\sum\nolimits_{1 \le i \le m} {F(G_{2} ,g_{1i} ) + } \sum\nolimits_{1 \le j \le n} {F(G_{1} ,g_{2i} )} }}{m + n}$$

where $g_{1i}$ is the gene of $G_{1}$. m denotes the number of genes in $G_{1}$ and n denotes the number of genes in $G_{2}$.

Finally, by Eq. 4, we could obtain the similarity between two diseases.

### Encoding method

Firstly, we searched diseases that cause PTSA in PubMed. Then, we obtained more diseases which are related to these disease by DO. We totally found 23 diseases which could cause PTSA and these diseases are related to 2387 kinds of diseases in DO. Then, we found these diseases are corresponded to 6875 kinds of proteins by Uniprot. These proteins could be the features of each disease.

The encoding method is as following:

5 $$F_{d} = \{ P_{1} ,P_{2} ,\ldots,P_{n} \}$$

where $F_{d}$ is the feature of disease. $P_{1}$ denotes whether this protein is related to this disease. If this protein is related to this disease according to Uniprot, *P*_1_ = 1, otherwise *P*_1_ = 0. n is the number of proteins we used.

Since we totally obtained 6875 proteins, n should be 6875. However, the dimension of features would be huge. Therefore, 523 most common proteins were selected as features since they are associated with at least 100 diseases. Finally, n should be 523 in our method. Therefore, each disease has a feature whose dimension is 1*523.

By the process above, we could build a disease network by the similarity of diseases and features of disease. In this network, each node is a disease and each edge is the similarity between two diseases. Therefore, there are 2387 nodes in the network, and each node contains the features of this disease. Then, GCN was used to encode the network.

For a given graph G = (V, E), V denotes the nodes and E denotes the edges. GCN is aim to use a nonlinear function to transfer network to output.

6 $$H^{(l + 1)} = f(H^{(l)} ,A)$$

$H^{(0)} = X$, which is the feature of the nodes.

Firstly, we need to obtain the Laplace matrix L:

7 $$L = D - A$$

D is the degree matrix, which could be calculated by Adjacency matrix A.

8 $${\hat{\text{D}}}_{{{\text{ii}}}} { = }\sum\nolimits_{j} {{\hat{\text{A}}}_{ij} }$$

D is a diagonal matrix. Then, we need to normalize L as following:

9 $$L^{sym} = D^{{ - \frac{1}{2}}} LD^{{ - \frac{1}{2}}} = I - D^{{ - \frac{1}{2}}} AD^{{ - \frac{1}{2}}}$$

The element of $L^{sym}$ is defined as

10 $$L_{i,j}^{sym} = \left\{ {\begin{array}{*{20}l} 1 \hfill & {i = j\;and\;\deg (v_{i} ) \ne 0} \hfill \\ { - \frac{1}{{\sqrt {\deg (v_{i} )\deg (v_{j} )} }}} \hfill & {i \ne j\;and\;v_{i} \;adjacent\;to\;v_{j} } \hfill \\ 0 \hfill & {otherwise} \hfill \\ \end{array} } \right.$$

With the Laplace matrix L, we can perform spectral convolution on the graph. In order to overcome the underfitting caused by too many parameters, some scholars have proposed a ‘Chebyshev’ method. In this method, filter function is:

11 $$g_{{\theta^{\prime}}} (\Lambda ) \approx \sum\limits_{k = 0}^{K} {\theta^{\prime}_{k} T_{k} (\tilde{\Lambda })}$$

where $\tilde{\Lambda } = \frac{2}{{\lambda_{\max } }}\Lambda - I_{N}$ $\theta_{k}^{^{\prime}}$ represents a Chebyshev vector. The definition of Chebyshev polynomial is as following:

12 $$T_{k} (x) = 2xT_{k - 1} (x) - T_{k - 2} (x)$$

where $$T_{0} (x) = 1$$, $$T_{1} (x) = x$$.

If we let $\lambda_{\max }$ = 2, K = 1, the first-order linear approximation of spectral convolution would be:

13 $$g_{{\theta^{\prime}}} *x \approx \theta^{\prime}_{0} x + \theta^{\prime}_{1} (L - I_{N} )x = \theta^{\prime}_{0} x - \theta^{\prime}_{1} D^{{ - \frac{1}{2}}} AD^{{ - \frac{1}{2}}} x$$

Therefore, the output of GCN would be:

14 $$H^{(l + 1)} = \sigma (D^{{ - \frac{1}{2}}} AD^{{ - \frac{1}{2}}} H^{(l)} W^{(l)} )$$

Overall, after encoding by GCN, each disease not only contains their protein features, but also its relationship with other diseases.

### Classification by Xgboost

Xgboost was proposed by Tianqi Chen [25]. The main advantage of using Xgboost in our work is the input could be sparse matrix. Since our feature is very sparse, Xgboost could handle these features.

Since Xgboost is derived from Gradient Boosting Decision Tree (GBDT) [26], we firstly introduced the workflow of GBDT.

**Table:** 

Algorithm: GBDT
Input: Train set $\{ x_{i} ,y_{i} \}_{{}}^{N}$, $y_{i} \in \{ - 1,1\}$ and Number of leaf nodes: J
Output: Model of GBDT $F(x)$
Initialization:$$F_{0} (x) = \frac{1}{2}\log \frac{{1 + \overline{y}}}{{1 - \overline{y}}}$$
For m = 1 to M do:
Calculate the training set sample gradient:
$$\overset{\lower0.5em\hbox{$\smash{\scriptscriptstyle\frown}$}}{y}_{i} = - \frac{{\partial L(y_{i} ,F(x_{i} ))}}{{\partial F(x_{i} )}}$$
According to the train set $\{ x_{i} ,y_{i} \}_{{}}^{N}$,build a CART regression tree:
$\{ R_{jm} \}^{J}$, $R_{jm}$ is the j_th_ feature space
Calculate the regression value for each leaf node:
$$r_{jm} = \frac{{\sum {_{{x_{i} \in R_{jm} }} \overset{\lower0.5em\hbox{$\smash{\scriptscriptstyle\frown}$}}{y}_{i} } }}{{\sum {_{{x_{i} \in R_{jm} }} } \left| {\overset{\lower0.5em\hbox{$\smash{\scriptscriptstyle\frown}$}}{y}_{i} } \right|(2 - \left| {\overset{\lower0.5em\hbox{$\smash{\scriptscriptstyle\frown}$}}{y}_{i} } \right|)}}$$
Obtain the Model:
$$F_{m} (x) = F_{m - 1} (x) + \sum\limits_{j = 1}^{J} {r_{jm} I(x \in R_{jm} )}$$
end

The objective function is consisted by two parts: loss function and regularization term.

15 $$Obj(\Theta ) = L(\theta ) + \Omega (\Theta )$$

$L(\theta )$ is the loss function and $\Omega (\Theta )$ denotes regularization function.

If T trees are trained, the model could be built as following:

16 $$\overset{\lower0.5em\hbox{$\smash{\scriptscriptstyle\frown}$}}{y}_{i} = \sum\limits_{t = 1}^{T} {f_{t} (x_{i} )}$$

Both Xgboost and GBDT’s basic classifier is CART, so the objective function could be as following:

17 $$Obj(\Theta ) = \sum\limits_{i}^{n} {l(y_{i} ,\overset{\lower0.5em\hbox{$\smash{\scriptscriptstyle\frown}$}}{y}_{i} )} + \sum\limits_{t = 1}^{T} {\Omega (f_{t} )}$$

Obtaining $f_{i}$ is our target. We trained the t_th_ tree based on the previous (t − 1) trees.

18 $$\begin{aligned} & \overset{\lower0.5em\hbox{$\smash{\scriptscriptstyle\frown}$}}{y}_{i}^{0} = 0, \\ & \overset{\lower0.5em\hbox{$\smash{\scriptscriptstyle\frown}$}}{y}_{i}^{1} = f_{1} (x_{i} ) = \overset{\lower0.5em\hbox{$\smash{\scriptscriptstyle\frown}$}}{y}_{i}^{0} + f_{1} (x_{i} ), \\ & \overset{\lower0.5em\hbox{$\smash{\scriptscriptstyle\frown}$}}{y}_{i}^{2} = f_{1} (x_{i} ) + f_{2} (x_{i} ) = \overset{\lower0.5em\hbox{$\smash{\scriptscriptstyle\frown}$}}{y}_{i}^{1} + f_{2} (x_{i} ), \\ & \vdots \\ & \overset{\lower0.5em\hbox{$\smash{\scriptscriptstyle\frown}$}}{y}_{i}^{2} = \sum\limits_{k = 1}^{t} {f_{k} (x_{i} )} = \overset{\lower0.5em\hbox{$\smash{\scriptscriptstyle\frown}$}}{y}_{i}^{t - 1} + f_{t} (x_{i} ), \\ \end{aligned}$$

Therefore, the t_th_ objective function is:

19 $$Obj^{(t)} = \sum\limits_{i}^{n} {l(y_{i} ,\overset{\lower0.5em\hbox{$\smash{\scriptscriptstyle\frown}$}}{y}^{t}_{i} )} + \sum\limits_{i = 1}^{t} {\Omega (f_{i} )}$$

Then, the loss function would be:

20 $$\begin{aligned} Obj^{(t)} & = \sum\limits_{i}^{n} {\left( {l(y_{i} ,\overset{\lower0.5em\hbox{$\smash{\scriptscriptstyle\frown}$}}{y}_{i}^{t - 1} ) + g_{i} f_{t} (x_{i} ) + \frac{1}{2}h_{i} f_{t}^{2} (x_{i} )} \right)} + \Omega (f_{t} ) \\ &\quad + \,{\text{constant}} \\ \end{aligned}$$

To obtain regularization term, decision tree could be defined as:

21 $$f_{t} (x) = w_{q(x)} ,w \in R^{M} ,q:R^{d} \to \{ 1,2, \ldots ,M\}$$

where q() can decide the nodes of input sample. w denotes the scores of nodes.

Regularization term would be obtained:

22 $$\Omega (f) = \gamma M + \frac{1}{2}\lambda \sum\limits_{j = 1}^{M} {w^{2}_{j} }$$

Both $\gamma$ and $\lambda$ are the parameters to control the complexity of the model.

So t_th_ tree’s objective function is as following:

23 $$\begin{aligned} Obj^{(t)} & \approx \sum\limits_{i = 1}^{n} {\left( {g_{i} w_{q} (x_{i} ) + \frac{1}{2}h_{i} w_{q}^{2} (x_{i} )} \right)} + \gamma M + \frac{1}{2}\lambda \sum\limits_{j = 1}^{M} {w_{j}^{2} } \\ & = \sum\limits_{j = 1}^{M} {\left( {\left( {\sum {g_{i} } } \right)w_{j} + \frac{1}{2}\left( {\sum {h_{i} + \lambda } } \right)w_{j}^{2} } \right)} + \gamma M \\ \end{aligned}$$

We could define $G_{j} = \sum {g_{i} }$ and $H_{j} = \sum {h_{i} }$, then we get:

24 $$Obj^{(t)} = \sum\limits_{j = 1}^{M} {(G_{j} w_{j} + } \frac{1}{2}(H_{j} + \lambda )w^{2}_{j} ) + \gamma M$$

Here, $w_{j}$ is independent of other items, we could get the optimal score of j_th_ node and optimal obj.

25 $$w_{j}^{*} = \frac{{ - G_{j} }}{{H_{j} + \lambda }}$$

26 $$obj^{*} = - \frac{1}{2}\sum\limits_{j = 1}^{T} {\frac{{G^{2}_{j} }}{{H_{j} + \lambda }}} + \gamma T$$

Finally, we should make the trees split according to certain rules.

27 $$Gain = \frac{1}{2}\left( {\frac{{G_{L}^{2} }}{{H_{L} + \lambda }} + \frac{{G_{R}^{2} }}{{H_{R} + \lambda }} - \frac{{(G_{L} + G_{R} )^{2} }}{{H_{L} + H_{R} + \lambda }}} \right) - \gamma$$

## Data Availability

All the datasets used in this paper could be downloaded from https://disease-ontology.org/; https://geneontology.org/.

## Abbreviations

PTSA: Psychological trauma and social avoidance
GCN: Graph convolutional network
PTSD: Post-traumatic stress disorder
PTG: Posttraumatic growth
OMIM: Online Mendelian inheritance in man
DO: Disease ontology
LLS: Log likelihood score
LSTM: Gradient boosting decision tree
CBSM: Cognitive-behavioral stress management
SVM: Support vector machine
ANN: Artificial neural network
DNN: Deep neural network
RF: Random forest
